# Comparative genomic data of the Avian Phylogenomics Project

**DOI:** 10.1186/2047-217X-3-26

**Published:** 2014-12-11

**Authors:** Guojie Zhang, Bo Li, Cai Li, M Thomas P Gilbert, Erich D Jarvis, Jun Wang, The Avian Genome Consortium

**Affiliations:** China National GeneBank, BGI-Shenzhen, Shenzhen, 518083 China; Centre for Social Evolution, Department of Biology, University of Copenhagen, Universitetsparken 15, DK-2100 Copenhagen, Denmark; Centre for GeoGenetics, Natural History Museum of Denmark, University of Copenhagen, Øster Voldgade 5-7, 1350 Copenhagen, Denmark; Trace and Environmental DNA laboratory, Department of Environment and Agriculture, Curtin University, Perth, Western Australia 6102 Australia; Department of Neurobiology, Howard Hughes Medical Institute, Duke University Medical Center, Durham, NC 27710 USA; Department of Biology, University of Copenhagen, DK-1165 Copenhagen, Denmark; Princess Al Jawhara Center of Excellence in the Research of Hereditary Disorders, King Abdulaziz University, Jeddah, 21589 Saudi Arabia; Macau University of Science and Technology, Avenida Wai long, Taipa, Macau, 999078 China; Department of Medicine, University of Hong Kong, Hong Kong, China

**Keywords:** Avian genomes, Phylogenomics, Whole genome sequencing

## Abstract

**Background:**

The evolutionary relationships of modern birds are among the most challenging to understand in systematic biology and have been debated for centuries. To address this challenge, we assembled or collected the genomes of 48 avian species spanning most orders of birds, including all Neognathae and two of the five Palaeognathae orders, and used the genomes to construct a genome-scale avian phylogenetic tree and perform comparative genomics analyses (Jarvis et al. in press; Zhang et al. in press). Here we release assemblies and datasets associated with the comparative genome analyses, which include 38 newly sequenced avian genomes plus previously released or simultaneously released genomes of Chicken, Zebra finch, Turkey, Pigeon, Peregrine falcon, Duck, Budgerigar, Adelie penguin, Emperor penguin and the Medium Ground Finch. We hope that this resource will serve future efforts in phylogenomics and comparative genomics.

**Findings:**

The 38 bird genomes were sequenced using the Illumina HiSeq 2000 platform and assembled using a whole genome shotgun strategy. The 48 genomes were categorized into two groups according to the N50 scaffold size of the assemblies: a high depth group comprising 23 species sequenced at high coverage (>50X) with multiple insert size libraries resulting in N50 scaffold sizes greater than 1 Mb (except the White-throated Tinamou and Bald Eagle); and a low depth group comprising 25 species sequenced at a low coverage (~30X) with two insert size libraries resulting in an average N50 scaffold size of about 50 kb. Repetitive elements comprised 4%-22% of the bird genomes. The assembled scaffolds allowed the homology-based annotation of 13,000 ~ 17000 protein coding genes in each avian genome relative to chicken, zebra finch and human, as well as comparative and sequence conservation analyses.

**Conclusions:**

Here we release full genome assemblies of 38 newly sequenced avian species, link genome assembly downloads for the 7 of the remaining 10 species, and provide a guideline of genomic data that has been generated and used in our Avian Phylogenomics Project. To the best of our knowledge, the Avian Phylogenomics Project is the biggest vertebrate comparative genomics project to date. The genomic data presented here is expected to accelerate further analyses in many fields, including phylogenetics, comparative genomics, evolution, neurobiology, development biology, and other related areas.

**Electronic supplementary material:**

The online version of this article (doi:10.1186/2047-217X-3-26) contains supplementary material, which is available to authorized users.

## Data description

Here we presented the genomes of 48 bird species, representing 36 orders of birds, including all Neognathae and two of the five Palaeognathae orders, collected by the Avian Genome Consortium ( [1], full author list of the Consortium provided in Additional file 1 and data in GigaDB [2]). The Chicken, Zebra finch, and Turkey genomes (sequenced using the Sanger method) were collected from the public domain. Another three genomes, the Pigeon, Peregrine Falcon and Duck, have been published during the development of this project [3–5], and five genomes, the Budgerigar, Crested Ibis, Little Egret, Emperor and Adele penguins, are reported in companion studies of this project [6, 7]. The data downloads for the remaining 38 genomes are released here.

## Genome sequencing

Tissue samples were collected from multiple sources, with the largest contributions from the Copenhagen Zoo (Denmark) and the Louisiana State University (USA). Most DNA samples were processed and quality control performed at the University of Copenhagen (Dr. Gilbert’s lab, Denmark) and Duke University (Dr. Jarvis’ lab, USA). The collected samples were then used for constructing pair-end libraries and sequenced using Illumina HiSeq 2000 platforms at the BGI (China). For the high-coverage birds, multiple pair-end libraries with a series of up to 9 insert sizes (170 bp, 500 bp, 800 bp, 2 kb, 5 kb, 10 kb and 20 kb) were constructed for each species, as part the first 100 species of the G10K project. For four birds (*Anas platyrhynchos*, *Picoides pubescens*, *Ophisthocomus hoazin* and *Tinamus guttatus*), libraries of some insert sizes were not constructed due to limited sample amounts or the sequencing strategies applied to those species. In addition, for the budgerigar genome, Roche 454 longer reads of multiple insert sizes were used [6]. For the low-coverage genomes, libraries of two insert sizes (500 bp and 800 bp) were constructed. The sequencing depths for high-coverage genomes were 50X to 160X, whereas the sequencing depths for low-coverage genomes were 24X to 39X. An effort was made to obtain DNA samples from tissues with associated museum voucher specimens with high quality metadata.

## Genome assembly

Before assembly, several quality control steps were performed to filter the low-quality raw reads. The clean reads of each bird were then passed to SOAPdenovo v1.05 [8] for *de novo* genome assembly. We tried different k-mers (from 23-mer to 33-mer) to construct contigs and chose the k-mer with the largest N50 contig length. In addition, we also tried different cut-offs of read pairs for different libraries to link contigs into scaffolds. The assembly with the largest N50 length was finally used.

All the assemblies have similar genome sizes, ranging from 1.04-1.26Gb (Table 1). The high-coverage genomes have a N50 scaffold length of >1 Mb, except for the White-throated Tinamou (*Tinamous guttatus*) with a scaffold N50 of 242 Kb and Bald Eagle (*Haliaeetus leucocephalus*) with a scaffold N50 of 670 Kb, due to no 10 kb and 20 kb libraries for these two genomes. For low-coverage genomes, the scaffold N50 lengths ranged from 30 kb to 64 kb. The N50 contig lengths for high-coverage genomes were from 19 kb to 55 kb, and the low coverage genomes were from 12 kb to 20 kb. The Parrot and Ostrich genomes were further assembled with the aid of optical mapping data, thus achieving much larger scaffold N50 sizes.

**Table 1:** **Basic statistics for the assemblies of avian species**

Species	Common name	Sequencing depth	Library	Assembly (contig/scaffold N50;total length)
**Published (Sanger sequencing)**				
***Gallus gallus***	Chicken	7X	-	36 K/7.07 M;1.05G
***Taeniopygia guttata***	Zebra finch	6X	-	39 K/10 M;1.2G
***Meleagris gallopavo***	Turkey	17X	-	12.6 K/1.5 M;1.04G
**High** **-** **coverage genomes**				
***Anas platyrhynchos domestica***	Peking duck	50X	200,500,2 k,5 k,10 k	26 K/1.2 M;1.1G
***Columba livia***	Pigeon	63X	200,500,800,2 k,5 k,10 k,20 k	22 K/3.2 M;1.11G
***Falco peregrinus***	Peregrine falcon	105X	200,500,800,2 k,5 k,10 k,20 k	28 K/3.9 M;1.18G
***Pygoscelis adeliae***	Adelie penguin	60X	200,500,800,2 k,5 k,10 k,20 k	19 K/5.0 M;1.23G
***Aptenodytes forsteri***	Emperor penguin	60X	200,500,2 k,5 k,10 k,20 k	30 K/5.1 M;1.26G
***Nipponia nippon***	Crested ibis	105X	200,500,800,2 k,5 k,10 k,20 k	22 K/5.4 M;1.17G
***Egretta garzetta***	Little egret	74X	200,500,800,2 k,5 k,10 k,20 k	24 K/3.1 M;1.2G
***Calypte anna***	Anna's hummingbird	110X	200,500,800,2 k,5 k,10 k,20 k	23 K/4 M;1.1G
***Chaetura pelagica***	Chimney swift	103X	200,500,800,2 k,5 k,10 k,20 k	27 K/3.8 M;1.1G
***Charadrius vociferus***	Killdeer	100X	200,500,800,2 k,5 k,10 k,20 k	32 K/3.6 M;1.2G
***Cuculus canorus***	Common cuckoo	100X	200,500,800,2 k,5 k,10 k,20 k	31 K/3 M;1.15G
***Ophisthocomus hoazin***	Hoatzin	100X	200,500,800,2 k,5 k,10 k	24 K/2.9 M;1.14G
***Geospiza fortis***	Medium ground finch	115X	200,500,800,2 k,5 k,10 k,20 k	30 K/5.2 M;1.07G
***Manacus vitellinus***	Golden-collared manakin	110X	200,500,800,2 k,5 k,10 k,20 k	34 K/2.5 M;1.12G
***Melopsittacus undulatus***	Budgerigar	160X	200, 500, 800, 2 k, 5 k, 10 k	55 K/10.6 M;1.1G
***Picoides pubescens***	Downy woodpecker	105X	200,500,800,2 k,5 k,10 k	20 K/2 M;1.17G
***Struthio camelus***	Ostrich	85X	200,500,800,2 k,5 k,10 k,20 k	29 K/3.5 M;1.23G
***Tinamus guttatus***	White-throated tinamou	100X	200,500,800,2 k,5 k	24 K/242 K;1.05G
***Corvus brachyrhynchos***	American crow	80X	200,500,800,2 k,5 k,10 k,20 k	24 K/6.9 M;1.1G
***Haliaeetus leucocephalus***	Bald eagle	88X	300,400,3 k,8 k	10 K/670 K;1.26G
**Low** **-** **coverage genomes**				
***Antrostomus carolinensis***	Chuck-will's-widow	30X	500, 800	17 K/45 K;1.15G
***Cariama cristata***	Red-legged seriema	24X	500, 800	17 K/54 K;1.15G
***Colius striatus***	Speckled mousebird	27X	500, 800	18 K/45 k;1.08G
***Merops nubicus***	Carmine bee-eater	37X	500, 800	20 K/47 K;1.06G
***Gavia stellata***	Red-throated loon	33X	500, 800	16 K/45 K;1.15G
***Balearica regulorum***	Grey-crowned crane	33X	500, 800	18 K/51 K;1.14G
***Apaloderma vittatum***	Bar-tailed trogon	28X	500, 800	19 K/56 K;1.08G
***Phalacrocorax carbo***	Great cormorant	24X	500, 800	15 K/48 K;1.15G
***Phaethon lepturus***	White-tailed tropicbird	39X	500, 800	18 K/47 K;1.16G
***Phoenicopterus ruber ruber***	American flamingo	33X	500, 800	16 K/37 K;1.14G
***Podiceps cristatus***	Great-crested grebe	30X	500, 800	13 K/30 K;1.15G
***Fulmarus glacialis***	Northern fulmar	33X	500, 800	17 K/46 K;1.14G
***Tyto alba***	Barn owl	27X	500, 800	13 K/51 K;1.14G
***Tauraco erythrolophus***	Red-crested turaco	30X	500, 800	18 K/55 K;1.17G
***Cathartes aura***	Turkey vulture	25X	500, 800	12 K/35 K;1.17G
***Eurypyga helias***	Sunbittern	33X	500, 800	16 K/46 K;1.1G
***Mesitornis unicolor***	Brown mesite	29X	500, 800	18 K/46 K;1.1G
***Leptosomus discolor***	Cuckoo-roller	32X	200, 500, 800	19 K/61 K;1.15G
***Chlamydotis macqueenii***	MacQueen's Bustard	27X	500, 800	18 K/45 K;1.09G
***Pelecanus crispus***	Dalmatian pelican	34X	500, 800	18 K/43 K;1.17G
***Pterocles gutturalis***	Yellow-thoated sandgrouse	25X	500, 800	17 K/49 K;1.07G
***Acanthisitta chloris***	Rifleman	29X	500, 800	18 K/64 K;1.05G
***Buceros rhinoceros***	Rhinoceros hornbill	35X	500, 800	14 K/51 K;1.08G
***Nestor notabilis***	Kea	32X	500, 800	16 K/37 K;1.14G
***Haliaeetus albicilla***	White-tailed eagle	26X	500, 800	20 K/56 K;1.14G

## Repeat annotation

RepeatMasker [9] and RepeatModeler [10] were used to perform repeat annotations for the bird genomes. The overall annotated content of transposable elements (TE) range from within 2-9% of all bird genomes except Woodpecker (Table 2). These TEs include long interspersed nuclear elements [LINEs], short interspersed nuclear elements [SINEs], long-terminal repeat [LTR] elements and DNA transposons). The exception Woodpecker genome has a TE content of 22%, which reflects a larger number of LINE CR1 elements (18% of the genome).

**Table 2:** **Percentages of genome annotated as transposable elements (TEs)**

Species	LINE	SINE	LTR	DNA	RC	Unknown	Total
***Merops nubicus***	5.01	0.07	1.30	0.14	0.01	1.26	7.78
***Picoides pubescens***	18.20	0.05	0.89	0.17	0.00	2.84	22.15
***Buceros rhinoceros***	3.62	0.08	1.05	0.16	0.01	1.09	6.00
***Apaloderma vittatum***	5.97	0.12	1.31	0.23	0.01	0.82	8.44
***Leptosomus discolor***	2.93	0.12	1.32	0.19	0.01	1.88	6.45
***Colius striatus***	6.54	0.10	2.19	0.19	0.00	0.39	9.42
***Haliaeetus albicilla***	2.55	0.14	1.71	0.19	0.01	0.77	5.37
***Haliaeetus leucocephalus***	2.01	0.17	1.89	0.22	0.00	2.59	6.89
***Cathartes aura***	2.21	0.17	1.05	0.19	0.00	0.92	4.54
***Tyto alba***	2.64	0.13	1.79	0.19	0.01	0.74	5.49
***Geospiza fortis***	3.65	0.06	3.37	0.31	0.04	0.80	8.23
***Taeniopygia guttata***	3.79	0.06	4.11	0.32	0.02	1.39	9.68
***Corvus brachyrhynchos***	3.73	0.07	2.43	0.22	0.02	0.90	7.37
***Manacus vitellinus***	4.43	0.08	1.08	0.25	0.01	0.72	6.58
***Acanthisitta chloris***	6.38	0.10	1.46	0.21	0.01	0.56	8.72
***Melopsittacus undulatus***	6.49	0.08	1.97	0.20	0.01	0.45	9.19
***Nestor notabilis***	4.60	0.10	1.32	0.18	0.00	0.37	6.57
***Falco peregrinus***	3.09	0.15	1.27	0.28	0.00	0.71	5.50
***Cariama cristata***	3.51	0.18	0.91	0.20	0.00	0.69	5.49
***Egretta garzetta***	3.92	0.12	1.42	0.24	0.01	1.22	6.93
***Pelecanus crispus***	3.94	0.15	1.87	0.21	0.01	1.27	7.45
***Nipponia nippon***	3.69	0.13	1.22	0.29	0.01	0.83	6.16
***Phalacrocorax carbo***	3.95	0.16	1.29	0.21	0.00	0.62	6.23
***Aptenodytes forsteri***	2.41	0.20	1.17	0.26	0.00	1.46	5.50
***Pygoscelis adeliae***	3.31	0.20	1.32	0.26	0.00	0.95	6.04
***Fulmarus glacialis***	2.86	0.18	1.19	0.22	0.01	0.87	5.32
***Gavia stellata***	3.17	0.14	0.71	0.22	0.01	0.85	5.09
***Eurypyga helias***	4.61	0.10	1.60	0.15	0.00	0.46	6.92
***Phaethon lepturus***	3.91	0.12	1.71	0.22	0.00	1.48	7.44
***Ophisthocomus hoazin***	4.69	0.11	1.30	0.16	0.01	1.63	7.90
***Balearica regulorum***	3.35	0.14	1.51	0.24	0.01	0.83	6.08
***Charadrius vociferus***	4.53	0.13	1.12	0.20	0.01	1.05	7.03
***Calypte anna***	5.62	0.07	1.23	0.21	0.01	0.91	8.05
***Chaetura pelagica***	5.28	0.11	0.90	0.19	0.00	2.57	9.05
***Antrostomus carolinensis***	5.40	0.12	1.84	0.33	0.02	0.53	8.24
***Chlamydotis macqueenii***	3.97	0.17	1.40	0.23	0.00	0.57	6.35
***Tauraco erythrolophus***	2.76	0.09	1.80	0.16	0.01	3.83	8.64
***Cuculus canorus***	7.84	0.08	0.67	0.27	0.01	0.58	9.45
***Mesitornis unicolor***	4.62	0.09	1.38	0.38	0.01	1.03	7.51
***Pterocles gutturalis***	3.46	0.09	1.36	0.17	0.01	0.67	5.75
***Columba livia***	4.18	0.09	0.76	0.35	0.01	1.87	7.25
***Phoenicopterus ruber***	2.69	0.15	1.04	0.23	0.01	1.49	5.60
***Podiceps cristatus***	4.80	0.10	1.60	0.20	0.01	0.60	7.31
***Gallus gallus***	6.01	0.08	1.65	1.01	0.01	1.07	9.82
***Meleagris gallopavo***	5.40	0.05	1.11	0.82	0.00	0.52	7.90
***Anas platyrhynchos***	4.05	0.10	1.10	0.20	0.01	0.39	5.85
***Struthio camelus***	2.88	0.18	0.17	0.36	0.01	0.90	4.49
***Tinamus guttatus***	2.73	0.09	0.30	0.33	0.01	0.65	4.11

## Protein-coding gene annotation

We used the homology-based method to annotate genes, with gene sets of chicken, zebra finch and human in Ensembl release 60 [11]. Because the quality of homology-based prediction strongly depends on the quality of the reference gene sets, we carefully chose the reference genes for the annotation pipeline. The protein sequences of these three species were compiled and used as a reference gene set template for homology-based gene predictions for the newly assembled bird genomes. We aligned protein sequences of the reference gene set to each genome by TBLASTN and used Genewise [12] to predict gene models in the genomes. A full description of the homology-based annotations is in our comparative genomics paper [1]. All the avian genomes have similar coding DNA sequence (CDS), exon, and intron lengths (Table 3).

**Table 3:** **Statistics of protein-coding gene annotations of all the birds**

Species	Gene number	Mean gene length (kb)	Mean CDS length (bp)	Mean exon length (bp)	Mean intron length (bp)	Mean intergenic length (kb)
***Acanthisitta chloris***	14596	13.5	1242	158.6	1800	12
***Anas platyrhynchos domestica***	16521	17.8	1317	160.7	2298	42
***Antrostomus carolinensis***	14676	12.0	1177	164.1	1747	12
***Apaloderma vittatum***	13615	13.5	1247	160.8	1806	12
***Aptenodytes forsteri***	16070	20.9	1397	161.6	2546	56
***Balearica regulorum***	14173	13.8	1276	162.7	1828	11
***Buceros rhinoceros***	13873	13.5	1267	160.4	1767	11
***Calypte anna***	16000	18.5	1386	161.7	2264	47
***Cariama cristata***	14216	13.7	1249	161.8	1849	11
***Cathartes aura***	13534	10.8	1109	166.4	1716	10
***Chaetura pelagica***	15373	19.8	1411	161.0	2364	51
***Charadrius vociferus***	16860	19.1	1324	161.8	2482	52
***Chlamydotis macqueenii***	13582	12.9	1257	162.9	1734	10
***Colius striatus***	13538	12.4	1190	161.1	1754	11
***Columba livia***	16652	18.3	1363	161.0	2277	46
***Corvus brachyrhynchos***	16562	17.9	1363	161.1	2220	48
***Cuculus canorus***	15889	20.0	1400	160.7	2413	48
***Egretta garzetta***	16585	18.6	1274	160.7	2496	52
***Eurypyga helias***	13974	12.3	1193	163.9	1763	11
***Falco peregrinus***	16242	19.9	1403	160.7	2389	49
***Fulmarus glacialis***	14306	12.8	1230	163.0	1765	11
***Gallus gallus***	16516	21.1	1433	158.1	2437	48
***Gavia stellata***	13454	13.2	1250	162.1	1776	11
***Geospiza fortis***	16286	17.9	1362	160.1	2198	46
***Haliaeetus albicilla***	13831	14.2	1258	161.1	1903	12
***Haliaeetus leucocephalus***	16526	19.0	1359	160.7	2370	36
***Leptosomus discolor***	14831	13.9	1236	163.2	1926	14
***Manacus vitellinus***	15285	18.8	1392	159.7	2262	46
***Meleagris gallopavo***	16051	17.4	1305	158.0	2215	52
***Melopsittacus undulatus***	15470	19.8	1395	162.2	2415	52
***Merops nubicus***	13467	13.0	1224	162.1	1798	11
***Mesitornis unicolor***	15371	11.4	1169	163.6	1666	11
***Nestor notabilis***	14074	14.4	1307	160.1	1822	12
***Nipponia nippon***	16756	19.4	1358	161.2	2434	51
***Ophisthocomus hoazin***	15702	20.0	1336	162.1	2582	55
***Pelecanus crispus***	14813	11.9	1183	164.8	1740	11
***Phaethon lepturus***	14970	12.7	1220	163.9	1781	11
***Phalacrocorax carbo***	13479	13.5	1258	162.0	1810	11
***Phoenicopterus ruber***	14024	11.7	1179	165.3	1716	10
***Picoides pubescens***	15576	20.0	1390	161.7	2450	47
***Podiceps cristatus***	13913	10.4	1137	165.8	1583	8
***Pterocles gutturalis***	13867	12.8	1235	162.5	1757	11
***Pygoscelis adeliae***	15270	21.3	1392	160.3	2589	58
***Struthio camelus***	16178	19.5	1289	161.0	2601	54
***Taeniopygia guttata***	17471	21.4	1383	153.5	2493	53
***Tauraco erythrolophus***	15435	13.2	1200	164.0	1894	12
***Tinamus guttatus***	15788	14.7	1288	162.0	1934	25
***Tyto alba***	13613	13.8	1240	160.8	1871	12

## Syntenic-based orthlogous annotation

To obtain more accurate orthology annotations for phylogenetic analyses in [13], we re-annotated some genes of the Chicken and Zebra Finch based on synteny, thereby correcting errors in the annotations due to being annotated independently with different methods. We first ran bi-directional BLAST to recognize the reciprocal best hits (considered as pairwise orthologs) between our re-annotated chicken genome and each of the other genomes. Then we identified syntenic blocks by using pairwise orthologs as anchors. We only kept the pairwise orthologs with syntenic support. In addition, we also considered the genomic syntenic information inferred from the LASTZ genome alignments, and removed pairwise orthologs without genomic syntenic support. After the above filtering, all the remaining pairwise orthologs were combined into a merged list by using a chicken gene set as a reference. We also required each orthologous group to have members in at least 42 out of 48 avian species. Ultimately, we obtained a list of 8295 syntenic-based orthologs. We used the same methods to generate 12815 syntenic-based orthologs of 24 mammalian species. A full description of the synteny-based annotations is found in our phylogenomics paper [13].

## Sequence alignments

### Protein coding gene alignment

CDS alignments for all orthologous genes were obtained by two rounds of alignments. In order to preserve the reading frames of CDS, we aligned the amino acid sequences and then back translated them into DNA alignments. In the first round of alignment, SATé-Prank [14] was employed to obtain the initial alignments, which were used to identify the aberrant over-aligned and under-aligned sequences. The aberrant sequences were then removed, and the second round of alignment were performed by SATé-MAFFT [14] for the filtered sequences to create the final multiple sequence alignments. The default JTT model inside SATé [14] was used as we found it to fit the data best for most genes. We also used the same method to generate the alignments of mammalian orthologs. More details of the alignment are presented in Jarvis et al. [13].

### Whole genome alignment

Whole genome alignments are very useful for comparative analyses, so we generated a multiple genome alignment of all 48 bird species. Firstly, pairwise alignments for each two genomes (with repeats masked) were produced by LASTZ [15], using chicken as the reference genome. Next chainNet [16] was introduced to obtain improved pairwise alignments. Finally, we used MULTIZ [17] to merge the pairwise alignments into multiple genome alignments. Approximately 400 Mb of each avian genome made it into the final alignment result. Thereafter, the alignment was filtered for over- and under-aligned errors, and for presence in 42 of 48 avian species. The resultant alignment was about 322 Mb, representing about one third of each genome, suggesting a large portion of the genome has been under strong constraints after different bird species diverged from their common ancestor. More details of the alignment are presented in Jarvis et al. [13].

## dN/dS estimates

We deposit dN/dS estimates (ratio of non-synonymous versus synonymous substitution rates) of the protein coding genes from Zhang et al. [1]. The dN/dS ratios were estimated by PAML [18] program for the orthologs. Based on the CDS alignment of either protein coding data set, we used the one-ratio branch model to estimate the overall dN/dS ratios for each avian orthologous group and each mammalian orthologous group. In addition, to investigate the evolutionary rates in three major avian clades (Palaeognathae, Galloanserae and Neoaves), we used the three-ratio branch model, which estimated one identical dN/dS ratio for each clade. More details about dN/dS analyses are presented in Zhang et al. [1].

## DNA sequence conservation

The overall level of conservation at the single nucleotide level could be estimated by PhastCons [19] based on multiple sequence alignments (MSA). First, the four-fold degenerate sites were extracted from 48-avian MSA and were used to estimate a neutral phylogenetic model by phyloFit [20], which is considered as the non-conserved model in PhastCons; we then ran PhastCons to estimate the conserved model. The conservation scores were predicted based on non-conserved and conserved models. We also used this method to estimate the sequence conservation for the 18-way mammalian genome alignments from the University of California at Santa Cruz (UCSC). Additional details of genome conservation are presented in the comparative genomics paper [1].

## List of scripts used in avian comparative genome project

We also deposit the key scripts used in the avian comparative genome project in GigaDB [2], which include: 1) scripts for cleaning raw reads and assembling the genome using SOAPdenovo; 2) scripts for RepeatMasker and RepeatModeler repeat annotation; 3) scripts for homology-based protein-coding gene annotation and combining the gene annotation evidences into final gene sets; 4) scripts for generating whole genome alignment of multiple genomes; 5) scripts for running PAML to estimate branch model dN/dS ratios; 6) scripts for calculating conservation scores based on whole genome alignments and predicting highly conserved elements; 7) scripts for quantifying gene synteny percentages in birds and mammals; 8) scripts for identifying large segmental deletions from list of orthologous genes; 9) scripts for detecting gene loss in 48 avian genomes. We provide readme files in the script directories describing the usage of the scripts.

## Availability and requirements

Download page for scripts:

https://github.com/gigascience/paper-zhang2014

Operating system: Linux

Programming language: Perl, R, Python

Other requirements: Some pipelines need external bioinformatics software, for which we provided executable files in the directories.

License: GNU General Public License version 3.0 (GPLv3)

Any restrictions to use by non-academics: No

## Availability of supporting data

The NCBI BioProject/SRA/Study IDs for are listed in Additional file 2. Other data files presented in this data note are available in the *GigaScience* repository, GigaDB [2].

## Authors’ information

The full author list of Avian Genome Consortium is provided in Additional file 1.

## Electronic supplementary material

Additional file 1: **Author list of the Avian Genome Consortium and contribution information of each author.** (XLS 85 KB)

Additional file 2: **NCBI accession numbers and GigaDB DOI for each bird.** (XLS 46 KB)

## Abbreviations

CDS: Coding sequence
Gb: Giga base pair
Kb: Kilo base pair
LINE: Long interspersed nuclear elements
MSA: Multiple sequences alignment
TE: Transposable element.
